# Evolution of Dietary Diversity: Further Consideration of Contextual and Multidimensional Features

**DOI:** 10.1016/j.advnut.2024.100284

**Published:** 2024-08-13

**Authors:** Kirsten A Herrick, Jennifer L Lerman

**Affiliations:** 1 Risk Factor Assessment Branch, Epidemiology and Genomics Research Program, Division of Cancer Control and Population Sciences, National Cancer Institute, National Institutes of Health, Rockville, MD, United States; 2 Department of International Health, Johns Hopkins Bloomberg School of Public Health, Baltimore, MD, United States

Dietary diversity is often used as a proxy for nutritional adequacy [1]. But, as pointed out by Bolo et al. [2], in this volume of *Advances in Nutrition*, just counting the number of foods is a crude metric, and in high-income countries, increased dietary diversity has been associated with higher obesity and cardiometabolic risk. To address this flaw and standardize the definition, the authors call for an expanded definition of dietary diversity that includes food evenness (the relative proportion of foods) and food complementarity (nutritional dissimilarity of foods consumed over time), beyond the traditional definition that just considers food coverage (number of foods). Inclusion of these additional dimensions within dietary diversity has the potential to refine guidance around what to eat; however, it will require tradeoffs that result from the long-standing tension between simplicity (counting foods reported or selecting from a short, predefined list of foods) and complexity (collecting open-ended, detailed descriptions of foods and amounts, potentially linked to a nutrient database). But, there are other metrics that leverage complex assessment tools and database linkages to address the limits identified in diet diversity and incorporate the multidimensionality of diet by considering the overall dietary pattern [3,4]. A few examples of these food group-based metrics include the following: the Healthy Eating Index (HEI), the Alternative Healthy Eating Index, the Mediterranean Diet Score, the Dietary Approaches to Stop Hypertension score [4], the United States Healthy Food Diversity Index [5], and the 2018 World Cancer Research Fund/American Institute for Cancer Research score [6].

Delving into 1 of these metrics, a brief exploration of HEI-2020 will demonstrate the utility of this index to address the shortfalls of dietary diversity. HEI is a measure of overall diet quality, independent of quantity, which can be used to assess alignment with the Dietary Guidelines for Americans [7]. The dietary guidance that results from the quinquennial review and consideration of the scientific literature is evidence based and seeks to balance the nutritional needs with noncommunicable disease reduction. Dietary diversity is intrinsic to how the HEI is constructed. An optimal score requires balanced intake across 13 food groups, where no single food or commodity is required for a perfect score. Foods are not counted, but rather intakes are summed across food groups and scored per 1000 kcal consumed. The HEI total score can be used to represent overall diet quality, and the set of individual component scores can be examined separately, and collectively can be used to reveal a pattern [8]. Importantly, components of the score that should be limited for good health (sodium, refined grains, saturated fats, and added sugars) are reversed scored, so that lower consumption of these items contribute higher component scores to the total score—addressing one of the limitations identified by Bolo et al. [2] in current conceptualizations of dietary diversity.

The HEI is an example of diet quality or diet pattern measures; others take similar approaches with variations in components included and scoring algorithms used. However, all have limitations. Most are scored on the collection of detailed dietary intake information and require extensive nutrient databases to estimate food group intakes; gathering both pieces are time and resource intensive [9]. There is also unavoidable measurement error and the potential for bias [9]. Although HEI is built around 2 well-established tenants of diet quality: diet adequacy and protection of health against noncommunicable disease, the application of some measures, such as the HEI and the Alternative HEI, to global populations has been somewhat limited. Specifically, HEI was developed for the United States context, and the dietary patterns it assesses contain foods and food groups, which likely vary by other factors. For discussion of a new index, the Diet Quality Questionnaire, which has country-adapted questionnaires for >100 countries, consider the Global Diet Quality Project [10].

Although the HEI and other indices may capture the multidimensionality around what is eaten, there are additional features to consider. Dietary intake is influenced by several contextual factors beyond the amounts of food groups consumed, with layers (who, what, when, where, why, and how eating occurs) that have typically been considered independently. Dietary guidance in the United States has traditionally focused on what (and how much) to eat [11]. For the first time, the 2020 Dietary Guidelines Advisory Committee expanded the topics considered by the committee to include 5 questions on the frequency of eating, illuminating the layer related to when eating occurs. The importance of when eating occurs has been underscored by new research on time-restricted eating and intermittent fasting [12]. There is also increasing recognition that these layers also interact in complex ways [13], for example, where you eat may change what is eaten, such as at home, in the car, or at school. With the advent of machine learning and artificial intelligence, the ability to consider the multidimensional natures of dietary intake is on the horizon.

In considering these other factors, we also have an opportunity to embrace other aspects of dietary intake that have been considered tangentially. The reciprocal interaction between food choices on the environment and climate and between climate change on food availability and nutrient composition are a rapidly expanding area of research that should be considered in dietary diversity. For example, dietary diversity could consider 2 fruits, say a cantaloupe and a mango, and defer to the fruit that is locally produced compared with one that must travel days by air or sea to reach the consumer, emphasizing sustainability, boosting the local economy, and perhaps improving nutrient retention simultaneously. Other emerging aspects of diet that might be worth considering within the construct of dietary diversity are level of food processing, formulation [14], and food matrix [15].

In conclusion, just as Bolo et al. [2] urge researchers to consider expanding dietary diversity scores from simple counts to include more granularity related to evenness and complementarity, we advocate going further. In both high-income and developing countries, there are opportunities to not only consider dietary patterns more wholistically but also begin to explore contextual factors that might influence diet, dietary choices, and human and planetary health in large degrees.

## Author contributions

KAH and JLL drafted the manuscript; and both authors read and approved the final manuscript.

## Conflict of interest

The authors are United States federal government employees. The authors report no conflicts of interest.

## Funding

The authors reported no funding received for this study.

## Disclaimer

The opinions expressed by the authors are their own, and this material should not be interpreted as representing the official viewpoint of the United States Department of Health and Human Services, the National Institutes of Health, or the National Cancer Institute.
